# HEALTH PRIORITIES IDENTIFICATION FOR INDIVIDUALS LIVING WITH DEMENTIA AND THEIR CAREGIVERS

**DOI:** 10.1093/geroni/igae098.0649

**Published:** 2024-12-31

**Authors:** Jack Banks, Caroline Blaum

**Affiliations:** Chair; Discussant

## Abstract

“What matters” is the foundation for the Age-Friendly Health System Initiative and yet many clinicians have a difficult time addressing it with their patients. Patient Priorities Care (PPC) is an evidence-based approach that identifies health priorities by first eliciting health values of older adults with multiple chronic conditions, integrating values into health outcome goals, and describing the one-thing to focus on. Clinicians’ then consider patients’ conditions and current treatments to identify care options that align with the identified health priorities. Furthermore, care of persons living with dementia (PlwD) involves care partners, thus identifying what matters most to PlwD is dyadic in nature. Recent studies have examined dyadic decision-making using the PPC approach with PlwD and their care partners. This symposium will present results from three studies that use PPC across diverse cultural and clinical contexts and discuss the role of PPC to achieve better dementia care. The first presentation will describe how clinicians consider health priorities with dyads facing dementia and discuss how health priorities identification can help care partners become better decision makers for their family members with dementia and multimorbidity. The second presentation will describe the cultural adaptation process of the PPC approach for older Hispanics with multimorbidity and dementia. The third presentation will describe how PPC facilitates deprescribing among older adults with multimorbidity and dementia. The panel will provide suggestions on how to better identify health priorities with PlwD and their care partners and share resources to facilitate the adoption and implementation of PPC with these dyads.

